# MicroRNAs Associated with Metformin Treatment in the Diabetes Prevention Program

**DOI:** 10.3390/ijms25115684

**Published:** 2024-05-23

**Authors:** Kimberly A. Lewis, Benjamin M. Stroebel, Li Zhang, Bradley Aouizerat, Aras N. Mattis, Elena Flowers

**Affiliations:** 1 Department of Physiological Nursing, School of Nursing, University of California, 2 Koret Way, San Francisco, CA 94143, USA; benjamin.stroebel@ucsf.edu (B.M.S.); elena.flowers@ucsf.edu (E.F.); 2 Department of Epidemiology and Biostatistics, University of California, San Francisco, CA 94143, USA; li.zhang@ucsf.edu; 3 College of Dentistry, New York University, New York, NY 10010, USA; bea4@nyu.edu; 4 Department of Pathology, University of California, San Francisco, CA 94143, USA; aras.mattis@ucsf.edu; 5 Institute for Human Genetics, University of California, San Francisco, CA 94143, USA

**Keywords:** diabetes risk, diabetes prevention program, microRNA, metformin, post-transcription

## Abstract

The Diabetes Prevention Program (DPP) randomized controlled trial demonstrated that metformin treatment reduced progression to type 2 diabetes (T2D) by 31% compared to placebo in adults with prediabetes. Circulating micro-ribonucleic acids (miRs) are promising biomarkers of T2D risk, but little is known about their associations with metformin regimens for T2D risk reduction. We compared the change in 24 circulating miRs from baseline to 2 years in a subset from DPP metformin intervention (*n* = 50) and placebo (*n* = 50) groups using Wilcoxon signed rank tests. Spearman correlations were used to evaluate associations between miR change and baseline clinical characteristics. Multiple linear regression was used to adjust for covariates. The sample was 73% female, 17% Black, 13% Hispanic, and 50 ± 11 years. Participants were obese, normotensive, prediabetic, and dyslipidemic. Change in 12 miR levels from baseline to 2 years was significantly different in the metformin group compared with placebo after adjusting for multiple comparisons: six (let-7c-5p, miR-151a-3p, miR-17-5p, miR-20b-5p, miR-29b-3p, and miR-93-5p) were significantly upregulated and six (miR-130b-3p, miR-22-3p, miR-222-3p, miR-320a-3p, miR-320c, miR-92a-3p) were significantly downregulated in the metformin group. These miRs help to explain how metformin is linked to T2D risk reduction, which may lead to novel biomarkers, therapeutics, and precision health strategies.

## 1. Introduction

At least 88 million adults in the United States (US) are considered to be at high risk of developing diabetes [1]. The Diabetes Prevention Program (DPP) randomized controlled trial demonstrated that for people with prediabetes, metformin therapy reduced progression to type 2 diabetes (T2D) by 31% compared to placebo [2,3,4,5].

Metformin is a biguanide that prevents the liver from converting fats and amino acids into glucose, thus lowering blood glucose levels [6,7]. Metformin primarily acts to lower blood sugar levels by inhibiting hepatic gluconeogenesis, the process by which the liver produces glucose [6,7,8]. The mechanism of action is activation of AMP-activated protein kinase (AMPK), an enzyme that plays a crucial role in energy regulation; AMPK activation leads to reduced expression of genes involved in glucose production and increased glucose uptake by cells, thereby lowering blood sugar levels [7,8]. In muscle and fat cells, metformin also improves insulin sensitivity, enabling these tissues to remove glucose more effectively from the bloodstream [7]. The AMPK pathway is also involved in repair of DNA damage and regulation of cellular autophagy and apoptosis [9].

Circulating microRNAs (miRs) are promising biomarkers for T2D risk [10,11] and responses to risk reduction interventions, including pharmacologic therapies [10,12,13]. Prior studies identified associations between expression of miRs and metformin therapy [14,15,16,17,18]. In participants with T2D treated with metformin therapy, let-7e-5p, let-7f-5p, miR-21-5p, miR-24-3p, miR-26b-5p, miR-126-5p, miR-129-5p, miR-130b-3p, miR-146a-5p, miR-148a-3p, miR-152-3p, miR-194-5p, miR-99a-5p were significantly downregulated after 3 months of metformin therapy [14,19]. In another study, several miRs that were upregulated in participants with untreated T2D compared with healthy controls were then decreased following metformin therapy [16,20]. These miRs included let-7b-5p, miR-15a-5p, miR-15b-5p, miR-16-5p, miR-16-2-3p, miR-25-3p, miR-30b-5p, miR-106b-3p, miR-195-5p, miR-3613-5p, and miR-424-5p. In human endothelial cells exposed in vitro to long-term metformin, 27 miRNAs were differentially expressed including miR-100-5p, miR-125-5p, miR-654-3p, miR-217, and miR-216a-3p/5p [21]. Though miRs have been associated with metformin therapy for people who already have T2D, little is known about their associations with metformin therapy for T2D risk reduction in people with prediabetes. The purpose of this study is to evaluate a subset of participants from the DPP study to assess whether metformin therapy compared with placebo is associated with corresponding changes in miR expression.

## 2. Results

Sample and clinical characteristics by study group are detailed in Table 1. At baseline, 73% self-identified as female, 17% Black race, and 13% Hispanic ethnicity. Participants were a mean age of 50 ± 11 years. On average the participants were obese (BMI 33.6 ± 6.7 kg/m^2^), normotensive, prediabetic, and dyslipidemic. No significant differences were found between the sample characteristics, clinical characteristics (Table 1), or standardized miR levels (Table 2) of the metformin and placebo groups at baseline. Thirty percent of the placebo group developed T2D in 2 years compared to 24% of the metformin group. Adherence to the prescribed metformin medication regimen was high overall. Study staff estimated that the participants received ≥80% exposure for 77% of participants who identified as White race, 83% of participants who identified as Black race, 58% of participants who identified as Hispanic ethnicity, and 75% for participants who self-identified as “Other Race or ethnicity”.

**Figure 1:**
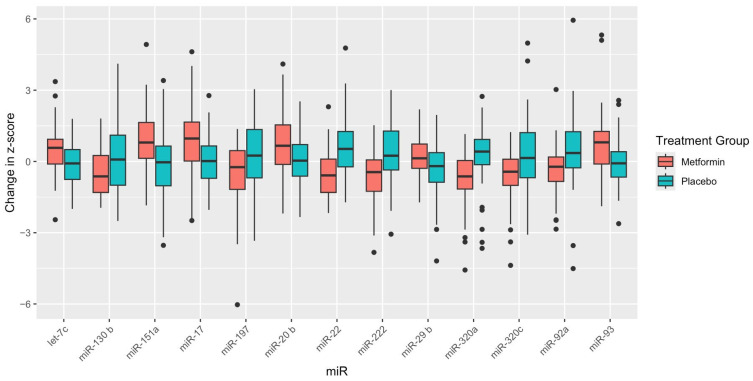
Box plots of mean change in MicroRNA expression level by treatment group for microRNAs significant after adjustment for multiple comparisons. Note: MiR-93a-5p was borderline statistically significant after adjusting for multiple comparisons (FDR = 0.059).

Table 2 reflects the baseline miR expression levels, 2-year miR expression levels, and mean change by treatment group, with both unadjusted and adjusted *p*-values to compare the differences between treatment groups. The change in 12 miRs was significantly different between treatment groups after 2 years and after FDR adjustment for multiple comparisons: let-7c-5p, miR-130b-3p, miR-151a-3p, miR-17-5p, miR-20b-5p, miR-22-3p, miR-222-3p, miR-29b-3p, miR-320a-3p, miR-320c, miR-92a-3p, miR-93-5p (Figure 1). Six miRs were significantly upregulated (i.e., let-7c-5p, miR-151a-3p, miR-17-5p, miR-20b-5p, miR-29b-3p, and miR-93-5p) and six miRs were significantly downregulated (i.e., miR-130b-3p, miR-22-3p, miR-222-3p, miR-320a-3p, miR-320c, and miR-92a-3p) in the metformin group after 2 years of therapy compared with placebo.

### 2.1. Predictors of miR Change after 2 Years

For the 12 miRs with adjusted *p* values less than 0.05, a multiple regression was applied to account for the influence of covariates (i.e., age, sex, race and ethnicity, and BMI) on mean change (Table 3). For seven of the adjusted miR models (i.e., let-7c-5p, miR-151a-3p, miR-222-3p, miR-29b-3p, miR-320a-3p, miR-320c, miR-92a-3p), treatment group was the only significant independent predictor of miR level. Age was a significant independent predictor of miR-130b-3p. Self-identified female gender was a significant independent predictor of miR-22-3p. Black race was a significant independent predictor of both miR-17-5p and miR-22-3p mean change and was of borderline significance for miR-20b-5p (adjusted *p* = 0.056). The “other race and ethnicity” category was a significant predictor of both miR-20b-5p and miR-93-5p z-scores in the regression model.

### 2.2. Correlations between Baseline Clinical Characteristics and Changes in MiRs

**Figure 2:**
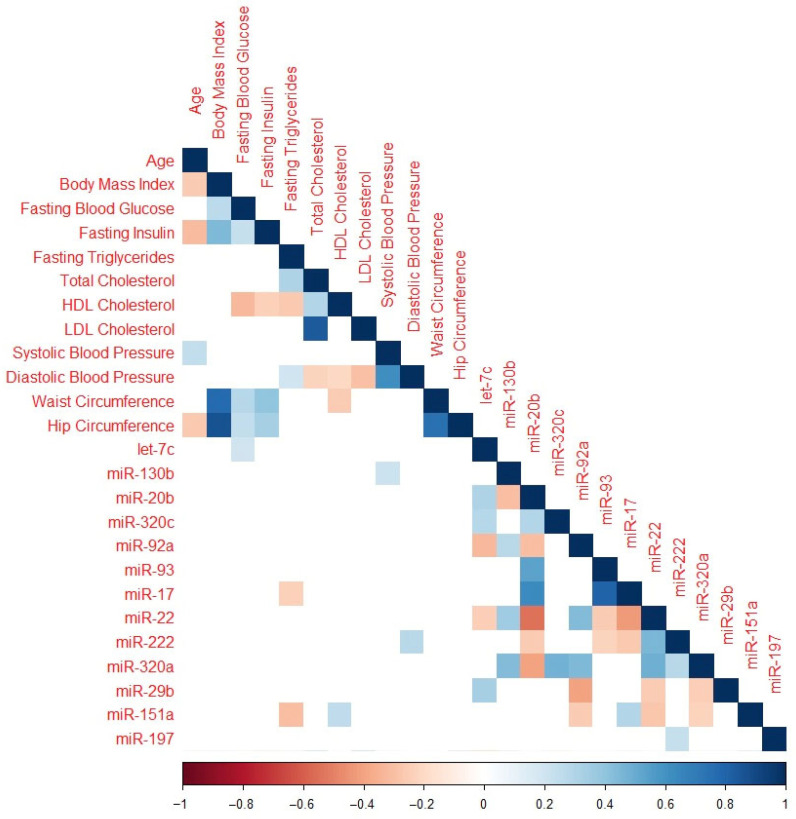
Correlation plot featuring only miRNAs significant after adjustment for multiple comparisons and baseline clinical characteristics. Cells with *p* > 0.05 are omitted. Color coding represents the Spearman rank correlation coefficient with red indicating an inverse association and blue indicating a positive association.

Significant correlations between baseline clinical characteristics and miR change in z-scores are presented in Figure 2. Baseline fasting blood glucose was inversely associated with mean change in let-7c-5p. Baseline fasting triglycerides were positively correlated with miR-130b-3p and miR-151a-3p. Total cholesterol was positively correlated with miR-20b-5p and negatively correlated with miR-22-3p. High-density lipoprotein (HDL) cholesterol was inversely associated with miR-151a-3p, while low-density lipoprotein (LDL) cholesterol was positively associated with miR-20b-5p. Systolic blood pressure and hip circumference were both inversely associated with miR-130b-3p, and diastolic blood pressure was inversely associated with miR-222-3p expression.

Table 4 summarizes what is known about the miRs, their response to metformin in our study, significant independent predictors or covariates in the multiple regression models, associations with clinical characteristics at baseline, gene targets, and mechanisms of action related to T2D risk.

## 3. Discussion

We compared mean change in standardized circulating miR expression levels after two years of metformin therapy versus placebo in adult participants in the Diabetes Prevention Program randomized controlled trial. The change in levels of twelve miRs was significantly different between treatment groups, six of which were upregulated and six of which were downregulated. Eight of these miRs are novel and have not previously been described in relation to metformin response (i.e., miR-151a-3p, miR-17-5p, miR-20b-5p, miR-22-3p, miR-320a-3p, miR-320c, miR-92a-3p, and miR-93-5p). Findings from the other four miRs—let7c-5p, miR-130b-3p, miR-222-3p, and miR-29b-3p—are aligned with the previously reported literature about metformin response.

Collectively, what is known about this set of miRs to date suggests that metformin therapy acts via myriad functions, organs, tissues, and biological systems related to the progression from an at-risk state to T2D disease onset, with or without development of related comorbidities. Differentially expressed miRs may regulate neuroinflammation, cellular senescence and aging, glucose and lipid metabolism, insulin signaling pathways, vasculitis and thrombogenesis, and immune function. Though the science is still developing, the findings from our study point to subgroups who may be of highest risk or receive the best therapeutic benefit from metformin therapy. These miRs provide mechanistic clues for optimal metformin treatment response, advancing our knowledge about precision prevention for T2D. Their diversity underscores the complexity of T2D prevention.

### 3.1. let-7c-5p

In our study, we observed downregulation of let-7c-5p in the metformin group after two years compared with controls. This observation aligns with previous findings about members of the let-7-5p family of miRNAs, such as let-7b-5p, let-7e-5p, and let-7f-5p, which were also reported to be downregulated after 3-12 months of metformin use [14,16,19,20]. After adjusting for covariates, the treatment group was the only significant predictor of let-7c-5p change after 2 years of therapy; however, the mean change was inversely correlated with baseline fasting blood glucose in our participants. This finding suggests that those with the highest blood glucose levels when they begin metformin therapy may see the greatest reduction in let-7c-5p levels over time.

Metformin’s beneficial effect may in part be due to let-7c-5p’s role in neuroinflammation [22]. A prior study of let-7c-5p in individuals with T2D and mild cognitive impairment (MCI) found elevated exosomal let-7c-5p levels in these people with T2D compared to those with T2D but without MCI. Zhang et al. (2022) observed a negative correlation between plasma interleukin (IL)-10 and exosomal let-7c-5p levels [22]. Given IL-10’s role in inhibiting neuroinflammation linked to diabetic cognitive dysfunction, the study suggests that let-7c-5p might exacerbate MCI in T2DM patients by reducing IL-10, affecting neuroinflammation pathways. For patients taking metformin, the downregulated let-7c-5p levels may allow IL-10 levels to normalize, thus inhibiting neuroinflammation in adults at risk for developing T2D.

The let-7 family of miRs is also known to target genes like the insulin receptor (*INSR*) and insulin receptor substrate 2 (*IRS2*), which are essential for insulin signaling. [23] Let-7c-5p specifically may regulate receptor tyrosine-protein kinase (*ERBB4*) and neuregulin-4 (*NRG4*) genes [24] as targets in obesity-associated metabolic disorders like T2D.

Let-7c has been linked to both metformin response and senescence and cellular aging [25]. In at least one mouse model study, metformin decreased cellular senescence by increasing DICER1 protein, lowering P16 and P21 protein levels, decreasing the abundance of inflammatory cytokines and oncogenes that are hallmarks of the senescence-associated secretory phenotype (SASP) [25]. Let-7c, along with miR-130b-3p, are two of the miRs identified in our study that are considered regulators of cellular senescence and influenced by metformin therapy [25,26].

### 3.2. miR-130b-3p

miR-130b-3p was significantly downregulated in the metformin therapy group in our study of at-risk individuals after 2 years. This finding was aligned with previous work by Demirsoy et al. (2018) in which miR-130b-3p was downregulated after 3 months of metformin therapy in people who had already developed T2D [19]. In another study of the participants of indigenous Native American descent, miR-130b-3p levels were upregulated in an increasing pattern based on glucose tolerance—from normal glucose tolerance (lowest miR-130b-5p levels) to impaired glucose tolerance to T2D (highest miR-130b-3p levels) [27].

Age was a significant independent predictor of miR-130b-3p change, with higher levels associated with older age in years. miR-130b-3p was positively correlated with baseline triglycerides and negatively correlated with baseline systolic blood pressure and hip circumference. Prabu et al. (2015) found links between miR-130b-3p and dyslipidemia in individuals who reported Asian Indian race group with impaired glucose tolerance versus with T2D [27]. Overexpression of miR-130b-3p has been linked to both diet-induced mouse models of diabetes [27] and high-fat diets in mice [28]. We found that triglyceride levels at baseline were significantly positively correlated with change in miR-130b-3p after 2 years. Overexpression of miR-130b-3p has been linked to increased triglyceride secretion into the bloodstream [29]. It may be that those individuals with higher levels of triglycerides at baseline are less likely to experience a reduction in miR-130b-3p with metformin therapy.

Elevated miR-130b-3p expression was associated with reduced intracellular pancreatic beta cell ATP levels in both rats and humans with T2D [30]. MiR-130b-3p is also thought to be a regulator of lipid homeostasis [29]. Overexpression of miR-130b in adipose tissue was associated with changed expression of critical genes implicated in lipid metabolism, with microsomal triglyceride transfer protein mRNA and protein expression levels increasing significantly in response to increased miR-130b-3p [29].

Ofori et al. (2017) demonstrated negative regulatory effects of miR-130b-3p on pyruvate dehydrogenase E1 alpha (PDHA1) and on glucokinase (GCK) proteins, which are both involved in ATP production [30]. In that study, overexpression of miR-130b-3p in the insulin-secreting INS-1 832/13 cell line resulted in altered dynamics of intracellular ATP/ADP ratio ultimately perturbing fundamental ATP-requiring beta cell processes such as glucose-stimulated insulin secretion, insulin biosynthesis and processing [30].

### 3.3. miR-151a-3p

MiR-151a-3p was upregulated in the metformin group after two years in our sample. To the best of our knowledge, no prior studies have reported on the relationship between miR-151a-3p and metformin therapy or its role in T2D. It may be related to atherosclerosis and cardiovascular comorbidity associated with T2D, however, because miR-151 may inhibit the apoptosis of endothelial cells in atherosclerosis and target IL-17A [31]. This may be characterizing complications from, rather than the etiology of, elevated fasting blood glucose and prediabetes. In our sample, miR-151a-3p was positively correlated with baseline fasting triglycerides and inversely correlated with baseline HDL, so its function may be related to cholesterol, but further investigation is needed.

### 3.4. miR-17-5p

MiR-17-5p is involved in cell proliferation, inflammation, mitochondrial function, and diabetes-related vascular damage [32]. Altered expression of miR-17-5p has previously been associated with T2D and gestational diabetes, obesity, and non-alcoholic fatty liver disease (NAFLD) [32,33], but it has not yet been reported in relation to metformin therapy for these diseases. miR-17-5p was upregulated in our metformin sample compared with placebo.

The direction of change (i.e., upregulated vs. downregulated) in previous studies has been mixed depending on the comparison group, the tissue type, and the specific disease pathology being studied. MiR-17-5p has been found to be upregulated in the blood of people with both T2D and NAFLD compared to people without NAFLD [34]. Upregulation of miR-17-5p also may be linked to the regulation of smooth muscle cell proliferation in the development of cardiovascular complications of T2D. It may play a role in the link between adipose tissue dysfunction and the development of obesity-associated disorders, including T2D, because upregulation has been significantly related to lower visceral fat mass, lower circulating parameters of chronic glycemia, and improved insulin sensitivity [35].

Black race was a significant independent predictor of miR-17-5p levels after adjusting for covariates in our sample. In a longitudinal study of metformin adherence and benefits in the overall DPP trial, Black race was associated with a higher incidence of T2D and was associated with lower metformin regimen adherence in the trial [36]. However, in the present study, participants who identified as Black were not overrepresented in the subset who had developed T2D in either group. Adherence was highest in participants in our sample who identified as Black and lowest in participants who identified as Hispanic. While miR-17-5p was not assessed, we previously reported differences in other miRs between race and ethnic groups included in the DPP trial [37]. Further studies are needed to validate these differences and determine if they provide mechanistic insights between the construct of race and ethnicity and risk for T2D.

MiR-17-5p has been linked to at least 1181 target genes associated with metabolic disease, including neuronal differentiation 1 (*NEUROD1*), leptin (*LEP*), leptin receptor (*LEPR*), uncoupling protein 3 (*UCP3*), sirtuin 1 (*SIRT1*), peroxisome proliferator activated receptor α (*PPARA*), and low-density lipoprotein receptor (*LDLR*) [33]. Due to its central role in metabolic disease pathophysiology, further study of miR-17-5p should involve complex models and consider the myriad implications of altered expression.

### 3.5. miR-20b-5p

MiR-20b-5p was upregulated in the metformin group in our study after 2 years. In adults with T2D, miR-20b-5p has been found to be upregulated in exosomes of T2D patients, which can be transferred to the cells of the vascular endothelium, where it targets wingless-related integration site family member 9b (wnt9b) signaling to inhibit wound healing and angiogenesis [38]. In a study of four men with T2D, miR-20b-5p was upregulated compared to age- and sex-matched men with normal glucose tolerance, but not significantly upregulated compared with the men in their study with prediabetes and at high risk for developing T2D [38].

Black race was a borderline significant (*p* = 0.056) independent predictor of miR-20b-5p change. Like miR-122, miR-20b-5p has been associated with NAFLD in people with T2D [34]. MiRMiR-20b-5p in skeletal muscle may impair glucose metabolism, downregulate gene sets associated with immune function (including interferon-α and interferon-γ response, tumor necrosis factor-α, IL-2-STAT5, and IL6–janus kinase (JAK)–STAT3 signaling pathways), and upregulate gene sets associated with cholesterol homeostasis, fatty acid metabolism, and mammalian target of rapamycin (mTOR) signaling pathways [39]. Overexpression of miR-20b-5p in human skeletal muscle cells suppressed expression of Ak strain transforming interacting protein (AKTIP), signal transducer and activator of transcription 3 (STAT3), and glycogen synthase and impaired insulin signaling [39].

### 3.6. miR-22-3p

One of the most abundant miRs in the liver, miR-22-3p is involved in the regulation of lipid and glucose metabolism with differential effects in specific organs [40,41,42]. Its role in T2D may be complex because both overexpression and underexpression have been linked to pathophysiological effects on T2D incidence, inflammation, obesity, NAFLD, and liver steatosis [40,41,42].

In the present study, miR-22-3p was significantly downregulated in the metformin group after 2 years compared to placebo. MiR-22-3p has not previously been described in relation to metformin therapy, but it has previously been studied in people who responded to thiazolidinedione treatment for insulin resistance compared to those who did not respond [43]. In that study by Flowers et al. (2015), miR-22-3p expression levels were higher at baseline in insulin resistant individuals compared to insulin-sensitive participants, and higher at baseline in those who responded to the thiazolidinedione treatment compared to those who did not respond, though they did not compare the change in miR over time after therapy [43].

Both female gender and Black race were significant, independent predictors of miR-22-3p change after 2 years in our study. Sex-based differences in gene expression have been observed in both healthy mice and mice with T2D [44], but they did not report on known targets of miR-22-3p. Research in both human and mouse models suggests that expression of miRs in males and females may be either consistently, differentially, or oppositely expressed in specific diseases (i.e., one sex is upregulated and the other sex is downregulated) [32,45]. More work is needed to understand sex-biased expression of the miRs in patients with T2D, and to differentiate the effects of biological sex from self-reported gender related to miR expression and risk for T2D.

A previous study of monocytes from individuals from varying race and ethnic groups found miR-22-3p to be inversely correlated with HDL cholesterol levels and positively associated with BMI, but those associations were not observed in our sample [41]. Gene targets of miR-22-3p are associated with cholesterol metabolism, such as stearoyl-CoA desaturase gene (*SCD*) which encodes stearoyl-CoA desaturase [41,46].

### 3.7. miR-222-3p

MiR-222-3p was downregulated in the metformin group compared with placebo after 2 years, and the miR change was inversely associated with baseline diastolic blood pressure in our study. MiRMiR-222-3p has previously been described as downregulated in response to metformin therapy [17,18], and it was found to play a role in the therapeutic effects of the T2D drug pioglitazone in a mouse model, acting on skeletal muscle tissue through a mechanism unrelated to the drug’s known PPARγ action [32,47].

Mechanistic investigations have pointed to the involvement of miR-222-3p levels in T2D, supported by its targeting of genes like O-6-Methylguanine-DNA Methyltransferase (*MGMT*), protein phosphatase 2 regulatory subunit B-α isoform (*PPP2R2A*), and reversion-inducing cysteine-rich protein with Kazal motifs (*RECK*) associated with T2D pathology [32,48,49].

### 3.8. miR-29b-3p

Circulating miR-29b has been observed to be upregulated in adults with T2D and may be a potential prognostic marker of diabetes risk [50]. Its role in T2D may be in mediating fibrosis, human insulin gene transcription, and circadian rhythms in β cells by targeting the period circadian regulator 3 (*Per3*) gene [50]. MiR-29b-3p was significantly upregulated after 2 years of metformin treatment in our sample of at-risk individuals.

Metformin, an endogenous competing RNA of the H19 imprinted maternally expressed transcript (*H19*) gene, reduced expression of H19 in animal models [51]. Both matrix metalloproteinase (MMP)-2 and MMP-9 are possible targets of miR-29b-3p. Chen et al. (2019) speculated that metformin may also reduce the expression of MMPs via the H19/miR-29b-3p signaling pathways [51].

### 3.9. miR-320a-3p

MiR-320a-3p has not previously been described with metformin therapy. MiR-320a-3p was downregulated in the metformin group compared with placebo in our sample. In another pharmacologic study, miR-320a-3p was increased in people with insulin resistance verse insulin sensitive, and this miR was inversely associated with response to thiazolidinediones, perhaps based on the overall positive association with insulin resistance and greater room for a therapeutic response [43]. There is some evidence for a role in T2D-related complications (i.e., miR-320a-3p has been associated with diabetic retinopathy [52], cardiotoxicity [53], and differences based on social determinants (i.e., miR-320a was inversely related to glycemic impairment in South Asians [54].

### 3.10. miR-320c

MiR-320c was not previously described with metformin therapy. It was significantly upregulated in the metformin group after 2 years compared with placebo in our sample. MiR-320c has been studied for its role in diabetic nephropathy pathogenesis due to its actions on the PI3K/AKT pathway [55]. *PTEN* is a gene target of miR-320c. Sun et al. (2022) observed that the function of miR-320c was reversed by downregulation of *PTEN*, and that inhibition of miR-320c could alleviate toxicity of HK-2 cells induced by hyperglycemia [55]. It has also been linked to β cell damage in mouse models of type 1 diabetes [44].

### 3.11. miR-92a-3p

MiRMiR-92a-3p was downregulated in the metformin group versus placebo, and our study is describing miR-92a-3p for the first time in relation to metformin response. Upregulated in fibrous plaques of early-stage atherosclerotic lesions [56]. Like miR-320a-3p, miR-92a-3p has been associated with diabetic retinopathy [52]. Significantly downregulated in extracellular vesicles in response to pioglitazone treatment relative to placebo in people with T2D [57]. These results were unchanged after inclusion of metformin as a cofactor in their analysis model [57].

### 3.12. miR-93-5p

In previous studies of patients with T2D, miR-93-5p expression levels were significantly lower than in healthy controls [58]. In the present study, after 2 years of metformin therapy, participants in the metformin group had significantly upregulated miR-93-5p compared to placebo. This change may reflect a normalization effect in response to the metformin for this miR.

MiR-93 may negatively regulate glucose transporter 4 (GLUT4) which is a dominant regulator of whole-body glucose homeostasis [59]. Like let-7c, miR-93 may also regulate *ERBB4* and *NRG4* gene expression [24]. *ERBB4* is involved in carbohydrate and lipid metabolism pathways while *NRG4* is associated with cardiac pathology [24]. Particularly for miR-93-5p, the presence of single-nucleotide polymorphisms (SNPs) in the seed region may affect the interaction between the two genes, so the presence of variants could lead to either gain or loss of function and should be studied further as a potential therapeutic target [24].

### 3.13. Limitations and Future Directions

This sample was limited to 100 randomly selected participants from the overall DPP study with miR expression levels from baseline and 2 years available. The findings should be validated with prospective samples large enough to support detailed subgroup analyses to fully understand the best candidates for metformin prevention therapy.

Minimal evidence is available about how miRs change in response to metformin therapy of varying durations. Further repeated measures, longitudinal studies are needed to optimize treatment response and to fully understand the mechanisms that support prevention of T2D occurrence after metformin therapy. Our study does not have a measure of when normal glucose was achieved and how that might affect miR expression levels in the metformin group. Future studies may also consider comparing miR expression levels in relation to varying metformin doses or regimens, monitoring time to achieve normal fasting blood glucose or hemoglobin A1C levels.

Our study did not investigate the effect of time on miR expression levels in both placebo and treatment group, and the role that metformin has, if any, on inhibiting cellular aging. Some literature suggests that there are anti-aging benefits of metformin in healthy individuals, but the available evidence suggests that its effects on lifespan primarily result from its antihyperglycemic action, enhancing insulin sensitivity, reduction of oxidative stress and protective effects on the endothelium and vascular function [60].

As miR expression reflects how an individual is adapting to its environment, data about social determinants and behavioral and environmental factors would help to provide context to the biological findings. This is particularly important to understanding differences in miR expression by self-identified race, as that reflects a social construct as opposed to a biological construct [37]. As seen in this study and others [61], lack of controlling for genetic ancestry, discretely from the social constructs of race and ethnicity, may be one reason for the lack of validation of relationships between miRs and risk for T2D [37,61].

## 4. Materials and Methods

The study was a secondary analysis of a subset of participants (*n* = 100) from the metformin and placebo arms of the DPP who agreed to additional longitudinal blood collection. All participants in the DPP provided informed consent and all experiments were conducted in alignment with the Declaration of Helsinki. Institutional review board (IRB) approval for the secondary analysis was provided by the University of California, San Francisco IRB. Participant data and biospecimens from the baseline and enrollment visits and the year 2 study visit were included.

### 4.1. Diabetes Prevention Program

The Diabetes Prevention Program was a longitudinal, three-arm randomized controlled trial study that compared an intensive lifestyle intervention, metformin, and placebo [2,62]. The study design, inclusion and exclusion criteria, and sample recruitment and characteristics have been described previously [63]. For the metformin group, metformin therapy was administered orally at 850 mg per dose taken once per day for the first month and then increased to 850 mg orally twice per day for the remainder of the study. Exceptions were made if gastrointestinal symptoms interfered with tolerance, in which case the drug was titrated over a longer period than one month. Placebo was given once per day for the placebo group for the first month, and then increased to twice per day for the duration of the study. Adherence to the medication regimen for both placebo and the metformin groups was assessed quarterly with pill counts and structured interviews. The proportion of participants who took at least 80 percent of the prescribed dose of the study medication was 77 percent in the placebo group and 72 percent in the metformin group over the entire study period (*p* < 0.001).

The study described in this article used existing demographic (i.e., age, sex, race and ethnicity, body mass index (BMI)) and baseline clinical data (i.e., total cholesterol, triglycerides, low-density lipoprotein cholesterol (LDL), high-density lipoprotein cholesterol (HDL), fasting blood glucose, blood pressure, fasting blood glucose, fasting insulin, and metformin regimen adherence) from the DPP trial and banked biospecimens from the National Institute of Diabetes, Digestive Disease, and Kidney Diseases biorepository [36,37].

### 4.2. Molecular Data Collection

Blood was collected by venipuncture into vacutainers with heparin [37]. The Fireplex Multiplex Circulating MicroRNA Assay (Abcam, Waltham, MA, USA) was used for direct quantification of 58 miRs from plasma collected with heparin. miRs were hybridized to complementary oligonucleotides covalently attached to encoded hydrogel microparticles. The bound target was ligated to oligonucleotide adapter sequences that serve as universal polymerase chain reaction priming sites. The miR–adapter hybrid models were then denatured from the particles, and reverse transcription polymerase chain reaction was performed using a fluorescent forward primer. Once amplified, the fluorescent target was rehybridized to the original capture particles and scanned on an EMD Millipore Guava 6HT flow cytometer (Merck KGaA Darmstadt, Germany).

Expression of individual miRs was normalized using the set of miR probes (i.e., miR-92a-3p, mir-93-5p, miR-17-5p) identified by the geNorm algorithm. Prior to analysis, a lower limit of quantification (LLOQ) equal to two times the minimal detectable difference for each miR was applied to the samples. Only miRs detected in ≥90% of the samples after filtering of sub-LLOQ values were retained for comparison, then converted to z-scores to account for batch effects between data collection timepoints. This resulted in 35 miRs that were retained for the analysis.

### 4.3. Data Analysis

The sample was characterized using descriptive statistics, and chi-squared or Wilcoxon signed rank test for independent samples were used to evaluate baseline categorical and continuous demographics, clinical characteristics, and baseline miRs between groups. Mean change in levels of 24 circulating miRs from baseline to 2 years were compared between the metformin intervention group (*n* = 50) and the placebo group (*n* = 50) using Wilcoxon signed rank test for independent samples, and then adjusted for multiple comparisons. The Benjamini–Hochberg false discovery rate (FDR) method was used to adjust for multiple comparisons (FDR < 0.05). Spearman’s rank correlation was used to assess relationships between the change in miRs from baseline to 2 years and the baseline continuous clinical variables, adjusted for multiple comparisons. Multiple linear regression was used to assess the influence of covariates (age, sex, race and ethnicity, and BMI) on differences in mean change by study group.

## 5. Conclusions

This study investigated the effects of a two-year metformin therapy versus placebo on the levels of miR expression in adult participants from the DPP randomized controlled trial. Changes in the expression of twelve specific miRs were identified, of which six were upregulated and six were downregulated in response to metformin treatment. Among these miRs, nine were previously not known to be associated with metformin therapy response. The findings suggested that metformin’s mechanisms of action are complex, involving a range of functions, tissues, and biological systems related to the transition from prediabetes to T2D, along with its associated comorbidities.

This study suggests that different miRs are involved in various aspects of metformin’s therapeutic effects on T2D risk, possibly indicating subgroups of individuals who could benefit the most from metformin treatment. These effects extend far beyond the glucose-lowering benefits and the AMPK pathway. However, the study has limitations, including the small sample size and lack of detailed social determinant and environmental data. Further research with larger samples and considering factors such as dose variability and duration of therapy would provide a more comprehensive understanding of miR responses to metformin treatment and its role in T2D prevention.

## Figures and Tables

**Table 1:** Demographics and clinical characteristics in the parent study overall and by study group.

	Overall *n* = 150	Metformin *n* = 50	Placebo *n* = 50	*p*
Demographic or Clinical Characteristics	*n* (%) or *M* (SD)	*n* (%) or *M* (SD)	*n* (%) or *M* (SD)	
Age (years)	51.0 (10.6)	50.9 (10.5)	49.8 (10.8)	0.485
Sex at Birth *				0.791
Male	41 (27.3)	15 (30.0)	12 (24.0)	
Female	109 (72.7)	35 (70.0)	38 (76.0)	
Race and Ethnicity * (%)				0.878
White	94 (62.7)	33 (66.0)	32 (64.0)	
Black	28 (18.7)	9 (18.0)	8 (16.0)	
Hispanic	22 (14.7)	6 (12.0)	7 (14.0)	
Other	6 (4.0)	2 (4.0)	3 (6.0)	
Postmenopause (females only)		21 (60)	16 (42)	0.080
Fasting Blood Glucose	106 (7.1)	106.2 (6.7)	106.3 (7.5)	0.769
Fasting Insulin	24.8 (13.9)	27.2 (16.2)	25.3 (12.5)	0.152
Fasting Triglyceride	164.5 (107.0)	173.4 (130.8)	171.9 (117.1)	0.426
Total Cholesterol	205.4 (38.4)	201.2 (37.9)	208.0 (35.8)	0.627
HDL Cholesterol	47.4 (13.4)	46.1 (10.7)	46.6 (14.1)	0.372
LDL Cholesterol	125.3 (33.0)	120.9 (32.7)	127.6 (32.0)	0.516
Waist (cm)	102.2 (14.5)	101.3 (13.9)	102.5 (16.9)	0.85
Hip Circumference (cm)	113.4 (17.1)	111.2 (12.7)	112.5 (11.3)	0.293
Systolic Blood Pressure (mm/Hg)	124.9 (16.2)	125.7 (16.4)	125.2 (15.1)	0.846
Diastolic Blood Pressure (mm/Hg)	77.9 (8.6)	78.3 (8.6)	77.4 (8.8)	0.869
Weight (kg)	91.2 (19.3)	90.0 (18.3)	93.9 (21.1)	0.489
Body Mass Index	33.3 (6.6)	33.1 (6.6)	34.2 (6.8)	0.412

* Self-reported; HDL = high-density lipoprotein; LDL = low-density lipoprotein.

**Table 2:** Median microRNA expression levels at baseline, year 2, and median 2-year change in miRNA z-scores by treatment group.

MicroRNA (miR)	Metformin Baseline Median (IQR)	Placebo Baseline Median (IQR)	Metformin Year 2 Median (IQR)	Placebo Year 2 Median (IQR)	Metformin Change in miR Median (IQR)	Placebo Change in miR Median (IQR)	*p*-Value	FDR *
let-7c-5p	−0.385 (0.734)	−0.364 (1.198)	0.100 (1.013)	−0.347 (1.104)	0.574 (1.043)	−0.082 (1.258)	0.004	0.018
let-7f-5p	−0.303 (0.943)	−0.277 (0.875)	−0.283 (0.966)	−0.510 (0.879)	0.129 (1.180)	−0.169 (0.987)	0.136	0.237
miR-106b-5p	−0.331 (0.942)	−0.261 (0.754)	−0.060 (0.722)	−0.254 (0.603)	0.386 (1.125)	0.099 (0.930)	0.114	0.221
miR-122-5p	−0.340 (0.724)	−0.347 (0.655)	−0.056 (0.540)	−0.284 (1.034)	0.166 (0.724)	−0.009 (0.733)	0.560	0.700
miR-126-3p	−0.136 (1.449)	−0.170 (0.913)	−0.024 (0.850)	0.051 (1.007)	−0.109 (1.377)	0.384 (1.023)	0.048	0.121
miR-130b-3p	0.127 (0.939)	0.086 (0.886)	−0.581 (0.693)	0.098 (1.674)	−0.628 (1.557)	0.082 (2.106)	0.007	0.025
miR-146a-5p	−0.113 (1.717)	−0.147 (1.298)	−0.271 (0.862)	−0.263 (1.645)	−0.122 (1.452)	0.124 (1.459)	0.161	0.268
miR-151a-3p	−0.485 (1.120)	−0.237 (1.819)	0.120 (1.424)	−0.485 (1.067)	0.798 (1.509)	−0.035 (1.671)	0.001	0.004
miR-151a-5p	−0.430 (1.281)	−0.111 (1.324)	−0.050 (1.449)	0.095 (1.158)	0.477 (2.150)	0.216 (1.556)	0.343	0.501
miR-151b	−0.282 (1.524)	−0.296 (1.427)	0.149 (1.344)	−0.120 (0.950)	0.540 (2.109)	0.099 (1.127)	0.111	0.221
miR-15a-5p	−0.138 (1.166)	−0.078 (1.016)	−0.249 (0.651)	−0.535 (1.320)	−0.066 (1.173)	−0.188 (0.956)	0.850	0.850
miR-15b-5p	−0.157 (0.962)	−0.020 (1.206)	0.129 (1.230)	−0.298 (0.986)	0.343 (1.774)	0.025 (1.393)	0.083	0.182
miR-16-5p	−0.202 (1.298)	0.102 (1.227)	0.055 (1.223)	−0.349 (1.410)	0.005 (1.425)	−0.382 (1.507)	0.074	0.172
miR-17-5p	−0.290 (1.099)	−0.112 (1.274)	0.693 (1.138)	−0.194 (0.881)	0.967 (1.641)	0.017 (1.361)	0.001	0.004
miR-192-5p	−0.129 (0.849)	−0.423 (0.823)	−0.077 (1.049)	−0.205 (0.942)	0.094 (0.961)	0.152 (1.261)	0.850	0.850
miR-20b-5p	−0.053 (0.994)	0.063 (1.182)	−0.236 (0.773)	0.280 (1.559)	−0.240 (1.632)	0.247 (2.035)	0.032	0.087
miR-21-5p	−0.339 (1.178)	−0.458 (1.013)	0.348 (1.734)	−0.324 (1.057)	0.662 (1.665)	0.034 (1.330)	0.015	0.045
miR-221-3p	−0.187 (1.061)	−0.194 (1.150)	−0.267 (0.898)	−0.480 (0.759)	0.001 (0.941)	−0.168 (1.396)	0.251	0.382
miR-222-3p	0.035 (1.110)	−0.060 (1.201)	−0.673 (0.852)	0.487 (1.387)	−0.586 (1.406)	0.528 (1.487)	0.000	0.000
miR-22-3p	−0.332 (1.164)	−0.234 (1.023)	−0.283 (1.060)	−0.065 (1.167)	0.220 (1.311)	−0.08 (1.301)	0.430	0.579
miR-23a-3p	−0.216 (1.191)	−0.290 (1.310)	−0.709 (0.632)	0.079 (1.119)	−0.448 (1.324)	0.243 (1.637)	0.000	0.001
miR-24-3p	−0.096 (1.214)	0.196 (1.572)	−0.271 (0.808)	−0.050 (1.116)	−0.212 (1.447)	−0.199 (1.924)	0.738	0.850
miR-27a-3p	−0.189 (1.243)	−0.124 (1.368)	0.019 (1.038)	0.010 (1.975)	0.281 (1.500)	0.061 (2.044)	0.785	0.850
miR-29b-3p	−0.340 (1.264)	−0.033 (1.271)	−0.295 (0.806)	−0.128 (1.327)	0.178 (1.681)	−0.116 (2.090)	0.376	0.526
miR-30a-5p	−0.430 (0.900)	−0.313 (0.896)	−0.155 (0.677)	−0.426 (1.007)	0.134 (1.022)	−0.196 (1.246)	0.012	0.039
miR-320a-3p	−0.451 (1.026)	−0.754 (1.026)	−0.369 (0.971)	−0.469 (1.035)	−0.012 (1.336)	−0.054 (0.916)	0.839	0.850
miR-320c	0.107 (1.008)	0.017 (1.005)	−0.593 (0.657)	0.421 (0.943)	−0.629 (1.201)	0.417 (1.064)	0.000	0.000
miR-342-3p	−0.072 (0.903)	−0.092 (0.916)	−0.569 (0.665)	−0.159 (1.134)	−0.431 (1.102)	0.146 (1.898)	0.007	0.025
miR-363-3p	−0.171 (1.246)	−0.356 (0.844)	−0.385 (0.922)	−0.240 (0.904)	−0.137 (0.995)	0.053 (1.187)	0.130	0.237
miR-486-3p	−0.558 (1.112)	−0.219 (0.920)	−0.271 (0.872)	−0.432 (0.996)	0.133 (1.056)	−0.016 (1.200)	0.497	0.644
miR-532-5p	−0.148 (1.101)	−0.706 (0.717)	−0.170 (0.710)	−0.339 (0.645)	0.204 (1.250)	0.109 (1.056)	0.834	0.850
miR-652-3p	−0.793 (0.951)	−0.288 (1.272)	−0.112 (0.878)	−0.204 (0.897)	0.446 (1.270)	−0.013 (1.611)	0.182	0.290
miR-92a-3p	−0.723 (1.345)	−0.723 (1.589)	−0.124 (1.349)	−0.107 (1.000)	0.228 (1.831)	0.194 (1.771)	0.728	0.850
miR-93-5p	−0.400 (1.025)	−0.176 (1.036)	−0.653 (0.779)	0.063 (1.096)	−0.222 (1.029)	0.356 (1.521)	0.001	0.004

FDR = Benjamini–Hochberg method false discovery rate adjustment for multiple comparisons, * significance set at adjusted *p* < 0.05.

**Table 3:** Multivariate linear regression of mean change in microRNA z-score by treatment group, adjusted for baseline age, sex, race and ethnicity, and body mass index (BMI).

	Metformin		Age		Sex—Female		Black Race		Hispanic Ethnicity		Race/Ethnicity (Other)		BMI	
MicroRNA (miR)	Coefficient	*p*-Value	Coefficient	*p*-Value	Coefficient	*p*-Value	Coefficient	*p*-Value	Coefficient	*p*-Value	Coefficient	*p*-Value	Coefficient	*p*-Value
let-7c-5p	0.589	**0.005**	−0.004	0.729	0.003	0.729	0.318	0.285	0.153	0.624	−0.612	0.216	0.002	0.912
miR-130b-3p	−0.759	**0.003**	0.029	0.021	0.357	0.216	0.162	0.358	−0.320	0.398	0.048	0.937	0.023	0.272
miR-151a-3p	1.020	**0.001**	0.012	0.412	−0.033	0.919	0.656	0.113	0.394	0.364	1.085	0.114	0.014	0.547
miR-17-5p	0.780	**0.002**	0.018	0.155	0.112	0.700	0.983	**0.008**	0.454	0.237	0.815	0.179	−0.026	0.222
miR-20b-5p	0.652	**0.013**	0.015	0.256	−0.319	0.288	0.721	0.056	0.404	0.307	1.424	**0.024**	−0.007	0.743
miR-222-3p	−1.040	**<0.001**	−0.007	0.536	0.150	0.578	−0.355	0.294	−0.096	0.787	−0.864	0.125	−0.016	0.414
miR-22-3p	−1.064	**<0.001**	0.015	0.178	0.606	**0.024**	−0.710	**0.034**	0.020	0.954	−0.798	0.148	0.010	0.610
miR-29b-3p	0.526	**0.011**	0.007	0.495	−0.081	0.733	0.163	0.582	0.181	0.560	−0.034	0.944	−0.025	0.156
miR-320a-3p	−0.973	**<0.001**	−0.008	0.534	0.145	0.612	0.011	0.974	0.105	0.781	−0.744	0.213	0.009	0.672
miR-320c	−0.874	**0.002**	0.000	0.989	−0.249	0.431	0.232	0.555	0.265	0.523	−0.297	0.649	0.01	0.670
miR-92a-3p	−0.807	**0.004**	0.002	0.909	0.340	0.279	0.056	0.886	−0.683	0.100	0.375	0.564	0.003	0.903
miR-93-5p	0.778	**0.003**	0.020	0.111	−0.097	0.739	0.636	0.082	0.138	0.719	1.242	**0.042**	−0.007	0.748

BMI = body mass index. Bold indicates statistical significance, *p* < 0.05.

**Table 4:** Summary of the 12 differentially Expressed miRs, response to metformin therapy, demographic and clinical associations, target genes and proteins, and summary of mechanisms in type 2 diabetes.

miRNA	Change in Metformin Group	Significant Predictors/Associations with Clinical Variables	Target Genes and Proteins	Mechanism Summary
let-7c-5p	Downregulated	Baseline fasting blood glucose	Insulin receptor (*INSR*), insulin receptor substrate 2 (*IRS2*), Erb-b2 receptor tyrosine kinase 4 (*ERBB4*), neuregulin 4 (*NRG4*), DICER1 protein, p16, p21,	May inhibit neuroinflammation. Targets genes involved in insulin signaling, metabolic disorders, and neuroinflammation pathways. Linked to cellular senescence by acting on proteins, inflammatory cytokines, and oncogenes associated with senescence-associated secretory phenotype (SASP)
miR-130b-3p	Downregulated	Systolic blood pressure	Genes implicated in lipid metabolism, microsomal triglyceride transfer protein mRNA and protein expression, pyruvate dehydrogenase E1 alpha (PDHA1) and glucokinase (GCK) proteins	Associated with dyslipidemia, insulin resistance, and pancreatic beta cell ATP levels
miR-151a-3p	Upregulated	Baseline fasting triglycerides, baseline HDL	Interleukin 17 (IL-17A)	May inhibit apoptosis of endothelial cells in atherosclerosis, possibly related to cholesterol regulation
miR-17-5p	Upregulated	Black race	Neuronal differentiation 1 (*NEUROD1*), leptin (*LEP*), leptin receptor (*LEPR*), uncoupling protein 3 (*UCP3*), sirtuin 1 (*SIRT1*), peroxisome proliferator activated receptor α (*PPARA*), low-density lipoprotein receptor (*LDLR*)	Associated with metabolic diseases, cardiovascular complications, and adipose tissue dysfunction
miR-20b-5p	Upregulated	Black race	interferon-α and interferon-γ response, tumor necrosis factor-α, interleukin (IL)2-STAT5, and IL6–janus kinase (JAK)–STAT3 signaling pathways), and upregulate gene sets associated with cholesterol homeostasis, fatty acid metabolism, and mammalian target of rapamycin (mTOR) signaling pathways, of Ak strain transforming interacting protein (AKTIP), signal transducer and activator of transcription 3 (STAT3), and glycogen synthase and impaired insulin signaling	Role in vascular endothelium, wound healing, angiogenesis inhibition, and glucose metabolism impairment
miR-22-3p	Downregulated	Female sex, black race	Genes associated with cholesterol metabolism, including stearoyl-CoA desaturase gene (*SCD*)	Role in lipid and glucose metabolism, insulin resistance, and cholesterol regulation
miR-222-3p	Downregulated	NA	Phosphatase and tensin homolog (*PTEN*), O-6-Methylguanine-DNA Methyltransferase (*MGMT*), protein phosphatase 2 regulatory subunit B-α isoform (*PPP2R2A*), and reversion-inducing cysteine-rich protein with Kazal motifs (*RECK*)	Role in diabetes, inflammation, obesity, NAFLD, and liver steatosis
miR-29b-3p	Upregulated	NA	Period circadian regulator 3 (*Per3*) gene, H19 imprinted maternally expressed transcript (*H19*) gene, matrix metalloproteinase (MMP)-2 and MMP-9	Role in T2D may be mediating fibrosis, human insulin gene transcription, and circadian rhythms in β cells, potential prognostic marker of deteriorating glucose tolerance
miR-320a-3p	Downregulated	NA	RNA polymerase III subunit D (*polr3d*) gene, insulin-like growth factor 1 (IGF-I) (proangiogenic factor)	Potential role in diabetic retinopathy, glycemic impairment, cardiotoxicity
miR-320c	Upregulated	NA	PI3K/AKT pathway, *PTEN*	Involved in diabetic nephropathy, PTEN targeting, β cell damage
miR-92a-3p	Downregulated	NA	NA	Linked to atherosclerosis, diabetic retinopathy, and extracellular vesicles’ response to pioglitazone
miR-93-5p	Upregulated	NA	Glucose transporter 4 (GLUT4), *ERBB4*, *NRG4*	Involved in glucose homeostasis, regulation of target genes related to glucose metabolism, neuroinflammation, and cardiac pathology

NA = not applicable or unknown.

## Data Availability

The original contributions presented in the study are included in the article further inquiries can be directed to the corresponding author/s.

## References

[1] Centers for Disease Control and Prevention (2020). National Diabetes Statistics Report, 2020.

[2] Aroda V.R., Knowler W.C., Crandall J.P., Perreault L., Edelstein S.L., Jeffries S.L., Molitch M.E., Pi-Sunyer X., Darwin C., Heck-man-Stoddard B.M. (2017). Metformin for diabetes prevention: Insights gained from the Diabetes Prevention Program/Diabetes Prevention Program Outcomes Study. Diabetologia.

[3] Diabetes Prevention Program Research Group (2019). Long-term Effects of Metformin on Diabetes Prevention: Identification of Subgroups That Benefited Most in the Diabetes Prevention Program and Diabetes Prevention Program Outcomes Study. Diabetes Care.

[4] Knowler W.C., Barrett-Connor E., Fowler S.E., Hamman R.F., Lachin J.M., Walker E.A., Nathan D.M., Diabetes Prevention Program Research Group (2002). Reduction in the incidence of type 2 diabetes with lifestyle intervention or metformin. N. Engl. J. Med..

[5] Madsen K.S., Chi Y., Metzendorf M.-I., Richter B., Hemmingsen B. (2019). Metformin for prevention or delay of type 2 diabetes mellitus and its associated complications in persons at increased risk for the development of type 2 diabetes mellitus. Cochrane Database Syst. Rev..

[6] Correia S., Carvalho C., Santos M.S., Seica R., Oliveira C.R., Moreira P.I. (2008). Mechanisms of action of metformin in type 2 diabetes and associated complications: An overview. Mini-Reviews Med. Chem..

[7] He L. (2020). Metformin and Systemic Metabolism. Trends Pharmacol. Sci..

[8] Long Y.C., Zierath J.R. (2006). AMP-activated protein kinase signaling in metabolic regulation. J. Clin. Investig..

[9] Szewczuk M., Boguszewska K., Kazmierczak-Baranska J., Karwowski B.T. (2020). The role of AMPK in metabolism and its influence on DNA damage repair. Mol. Biol. Rep..

[10] Flowers E., Allen I.E., Kanaya A.M., Aouizerat B.E. (2021). Circulating microRNAs are associated with variability in fasting blood glucose over 12-months and target pathways related to type 2 diabetes: A pilot study. Diabetes Vasc. Dis. Res..

[11] Flowers E., Asam K., Allen I.E., Kanaya A.M., Aouizerat B.E. (2022). Co-expressed microRNAs, target genes and pathways related to metabolism, inflammation and endocrine function in individuals at risk for type 2 diabetes. Mol. Med. Rep..

[12] Flowers E., Kanaya A.M., Fukuoka Y., Allen I.E., Cooper B., Aouizerat B.E. (2017). Preliminary evidence supports circulating microRNAs as prognostic biomarkers for type 2 diabetes. Obes. Sci. Pr..

[13] Flowers E., Aouizerat B.E., Kanaya A.M., Florez J.C., Gong X., Zhang L. (2022). MicroRNAs Associated With Incident Diabetes in the Diabetes Prevention Program. J. Clin. Endocrinol. Metab..

[14] Alimoradi N., Firouzabadi N., Fatehi R. (2021). Metformin and insulin-resistant related diseases: Emphasis on the role of microRNAs. Biomed. Pharmacother..

[15] Chien H.-Y., Lee T.-P., Chen C.-Y., Chiu Y.-H., Lin Y.-C., Lee L.-S., Li W.-C. (2015). Circulating microRNA as a diagnostic marker in populations with type 2 diabetes mellitus and diabetic complications. J. Chin. Med. Assoc..

[16] Ghai V., Kim T.-K., Etheridge A., Nielsen T., Hansen T., Pedersen O., Galas D., Wang K. (2019). Extracellular Vesicle Encapsulated MicroRNAs in Patients with Type 2 Diabetes Are Affected by Metformin Treatment. J. Clin. Med..

[17] Yaribeygi H., Katsiki N., Behnam B., Iranpanah H., Sahebkar A. (2018). MicroRNAs and type 2 diabetes mellitus: Molecular mechanisms and the effect of antidiabetic drug treatment. Metabolism.

[18] Zhou J.Y., Xu B., Li L. (2015). A New Role for an Old Drug: Metformin Targets MicroRNAs in Treating Diabetes and Cancer. Drug Dev. Res..

[19] Demirsoy I.H., Ertural D.Y., Balci Ş., Çınkır Ş., Sezer K., Tamer L., Aras N. (2018). Profiles of Circulating miRNAs Following Metformin Treatment in Patients with Type 2 Diabetes. J. Med. Biochem..

[20] Pedersen H.K., Gudmundsdottir V., Nielsen H.B., Hyotylainen T., Nielsen T., Jensen B.A.H., Forslund K., Hildebrand F., Prifti E., Falony G. (2016). Human gut microbes impact host serum metabolome and insulin sensitivity. Nature.

[21] Giuliani A., Londin E., Ferracin M., Mensà E., Prattichizzo F., Ramini D., Marcheselli F., Recchioni R., Rippo M.R., Bonafè M. (2020). Long-term exposure of human endothelial cells to metformin modulates miRNAs and isomiRs. Sci. Rep..

[22] Zhang J., Jia H., Xi J., Bai X. (2022). miR-140-5p Attenuates High-Glucose-Induced Endothelial-to-Mesenchymal Transition in Human Retinal Microvascular Endothelial Cells by Promoting Autophagy Through the Inhibition of TGF-β2/Smad3 Pathway. Di-Abetol. Metab. Syndr..

[23] Frost R.J.A., Olson E.N. (2011). Control of glucose homeostasis and insulin sensitivity by the *Let-7* family of microRNAs. Proc. Natl. Acad. Sci. USA.

[24] Vulf M., Bograya M., Komar A., Khaziakhmatova O., Malashchenko V., Yurova K., Sirotkina A., Minchenko A., Kirienkova E., Gazatova N. (2023). NGR4 and ERBB4 as Promising Diagnostic and Therapeutic Targets for Metabolic Disorders. Front. Biosci..

[25] Hooten N.N., Martin-Montalvo A., Dluzen D.F., Zhang Y., Bernier M., Zonderman A.B., Becker K.G., Gorospe M., Cabo R., Evans M.K. (2016). Metformin-mediated increase in DICER1 regulates microRNA expression and cellular senescence. Aging Cell.

[26] Abdelmohsen K., Gorospe M. (2015). Noncoding RNA control of cellular senescence. Wiley Interdiscip. Rev. RNA.

[27] Prabu P., Rome S., Sathishkumar C., Aravind S., Mahalingam B., Shanthirani C.S., Gastebois C., Villard A., Mohan V., Balasubramanyam M. (2015). Circulating MiRNAs of ‘Asian Indian Phenotype’ Identified in Subjects with Impaired Glucose Tolerance and Patients with Type 2 Diabetes. PLoS ONE.

[28] Zhang M., Zhou Z., Wang J., Li S. (2016). MiR-130b promotes obesity associated adipose tissue inflammation and insulin resistance in diabetes mice through alleviating M2 macrophage polarization via repression of PPAR-γ. Immunol. Lett..

[29] Zhang L., Li J., Li L., Gao J., Song D., Xu D., Wang X., Wang W., Cao L., Qin X. (2019). MiRNA-133a-5p regulates car-diomyocyte hypertrophy in diabetes through cytochrome c oxidase subunit 4 homologous gene. Eur. J. Heart Fail..

[30] Ofori J.K., Salunkhe V.A., Bagge A., Vishnu N., Nagao M., Mulder H., Wollheim C.B., Eliasson L., Esguerra J.L.S. (2017). Elevated miR-130a/miR130b/miR-152 expression reduces intracellular ATP levels in the pancreatic beta cell. Sci. Rep..

[31] Chen F., Ye X., Jiang H., Zhu G., Miao S. (2020). MicroRNA-151 Attenuates Apoptosis of Endothelial Cells Induced by Oxidized Low-density Lipoprotein by Targeting Interleukin-17A (IL-17A). J. Cardiovasc. Transl. Res..

[32] Lewis K.A., Chang L., Cheung J., Aouizerat B.E., Jelliffe-Pawlowski L.L., McLemore M.R., Piening B., Rand L., Ryckman K.K., Flowers E. (2023). Systematic review of transcriptome and microRNAome associations with gestational diabetes mellitus. Front. Endocrinol..

[33] Assmann T.S., Milagro F.I., Martínez J.A. (2019). Crosstalk between microRNAs, the putative target genes and the lncRNA network in metabolic diseases. Mol. Med. Rep..

[34] Ye D., Zhang T., Lou G., Xu W., Dong F., Chen G., Liu Y. (2018). Plasma miR-17, miR-20a, miR-20b and miR-122 as potential biomarkers for diagnosis of NAFLD in type 2 diabetes mellitus patients. Life Sci..

[35] Klöting N., Berthold S., Kovacs P., Schön M.R., Fasshauer M., Ruschke K., Stumvoll M., Blüher M. (2009). MicroRNA expression in human omental and subcutaneous adipose tissue. PLoS ONE.

[36] Walker E.A., Gonzalez J.S., Tripputi M.T., Dagogo-Jack S., Matulik M.J., Montez M.G., Tadros S., Edelstein S.L., DPP Research Group (2020). Long-term metformin adherence in the Diabetes Prevention Program Outcomes Study. BMJ Open Diabetes Res. Care.

[37] Flowers E., Kanaya A.M., Zhang L., Aouizerat B.E. (2022). The Role of racial and ethnic factors in microRNA expression and risk for type 2 diabetes. Front. Genet..

[38] Xiong Y., Chen L., Yan C., Zhou W., Endo Y., Liu J., Hu L., Hu Y., Mi B., Liu G. (2019). Circulating Exosomal miR-20b-5p Inhibition Restores Wnt9b Signaling and Reverses Diabetes-Associated Impaired Wound Healing. Small.

[39] Katayama M., Wiklander O.P.B., Fritz T., Caidahl K., El-Andaloussi S., Zierath J.R., Krook A. (2019). Circulating Exosomal miR-20b-5p Is Elevated in Type 2 Diabetes and Could Impair Insulin Action in Human Skeletal Muscle. Diabetes.

[40] Gjorgjieva M., Sobolewski C., Ay A.-S., Abegg D., de Sousa M.C., Portius D., Berthou F., Fournier M., Maeder C., Rantakari P. (2020). Genetic Ablation of MiR-22 Fosters Diet-Induced Obesity and NAFLD Development. J. Pers. Med..

[41] Parker D.C., Wan M., Lohman K., Hou L., Nguyen A.T., Ding J., Bertoni A., Shea S., Burke G.L., Jacobs D.R. (2022). Monocyte miRNAs Are Associated With Type 2 Diabetes. Diabetes.

[42] Thibonnier M., Esau C. (2020). Metabolic Benefits of MicroRNA-22 Inhibition. Nucleic Acid Ther..

[43] Flowers E., Aouizerat B.E., Abbasi F., Lamendola C., Grove K.M., Fukuoka Y., Reaven G.M. (2015). Circulating microRNA-320a and microRNA-486 predict thiazolidinedione response: Moving towards precision health for diabetes prevention. Metabolism.

[44] Liu G., Li Y., Zhang T., Li M., Li S., He Q., Liu S., Xu M., Xiao T., Shao Z. (2021). Single-cell RNA Sequencing Reveals Sexually Dimorphic Transcriptome and Type 2 Diabetes Genes in Mouse Islet β Cells. Genom. Proteom. Bioinform..

[45] Guo L., Zhang Q., Ma X., Wang J., Liang T. (2017). miRNA and mRNA expression analysis reveals potential sex-biased miRNA expression. Sci. Rep..

[46] Ding J., Reynolds L.M., Zeller T., Müller C., Lohman K., Nicklas B.J., Kritchevsky S.B., Huang Z., de la Fuente A., Soranzo N. (2015). Alterations of a Cellular Cholesterol Metabolism Network Are a Molecular Feature of Obesity-Related Type 2 Diabetes and Cardiovascular Disease. Diabetes.

[47] de Mendonca M., de Sousa E., da Paixao A.O., dos Santos B.A., Spagnol A.R., Murata G.M., Araujo H.N., de Lima T.I., Guimaraes D.S.P.S.F., Silveira L.R. (2020). MicroRNA miR-222 mediates pioglitazone beneficial effects on skeletal muscle of diet-induced obese mice. Mol. Cell. Endocrinol..

[48] Filardi T., Catanzaro G., Grieco G.E., Splendiani E., Trocchianesi S., Santangelo C., Brunelli R., Guarino E., Sebastiani G., Dotta F. (2022). Identification and validation of miR-222-3p and miR-409-3p as plasma biomarkers in gestational diabetes mellitus sharing validated target genes involved in metabolic homeostasis. Int. J. Mol. Sci..

[49] Goldsworthy M., Bai Y., Li C.-M., Ge H., Lamas E., Hilton H., Esapa C.T., Baker D., Baron W., Juan T. (2016). Haploinsufficiency of the insulin receptor in the presence of a splice-site mutation in *ppp2r2a* results in a novel digenic mouse model of type 2 diabetes. Diabetes.

[50] Dalgaard L.T., Sorensen A.E., Hardikar A.A., Joglekar M. (2022). The microRNA-29 family: Role in metabolism and metabolic disease. Am. J. Physiol. Physiol..

[51] Chen Z., Wei H., Zhao X., Xin X., Peng L., Ning Y., Wang Y., Lan Y., Zhang Q. (2019). Metformin treatment alleviates polycystic ovary syndrome by decreasing the expression of MMP-2 and MMP-9 via H19/miR-29b-3p and AKT/mTOR/autophagy signaling pathways. J. Cell. Physiol..

[52] Solis-Vivanco A., Santamaría-Olmedo M., Rodríguez-Juárez D., Valdés-Flores M., González-Castor C., Velázquez-Cruz R., Ramírez-Salazar E., García-Ulloa A.C., Hidalgo-Bravo A. (2022). miR-145, miR-92a and miR-375 Show Differential Expression in Serum from Patients with Diabetic Retinopathies. Diagnostics.

[53] Beuzelin D., Kaeffer B. (2018). Exosomes and miRNA-Loaded Biomimetic Nanovehicles, a Focus on Their Potentials Preventing Type-2 Diabetes Linked to Metabolic Syndrome. Front. Immunol..

[54] Flowers E., Gadgil M., Aouizerat B.E., Kanaya A.M. (2015). Circulating micrornas associated with glycemic impairment and progression in Asian Indians. Biomark. Res..

[55] Sun Y., Qu H., Song Q., Shen Y., Wang L., Niu X. (2022). High-glucose induced toxicity in HK-2 cells can be alleviated by inhibition of miRNA-320c. Ren. Fail..

[56] Karere G.M., Glenn J.P., Li G., Konar A., VandeBerg J.L., Cox L.A. (2023). Potential miRNA biomarkers and therapeutic targets for early atherosclerotic lesions. Sci. Rep..

[57] Lopez Y.O.N., Casu A., Kovacova Z., Petrilli A.M., Sideleva O., Tharp W.G., Pratley R.E. (2022). Coordinated regulation of gene expression and microRNA changes in adipose tissue and circulating extracellular vesicles in response to pioglitazone treatment in humans with type 2 diabetes. Front. Endocrinol..

[58] Saleh A.A., El-Hefnawy S.M., Kasemy Z.A., Alhagaa A.A., Nooh M.Z., Arafat E.S. (2022). Mi-RNA-93 and Mi-RNA-152 in the Diagnosis of Type 2 Diabetes and Diabetic Retinopathy. Br. J. Biomed. Sci..

[59] Esteves J.V., Enguita F.J., Machado U.F. (2017). MicroRNAs-Mediated Regulation of Skeletal Muscle GLUT4 Expression and Translocation in Insulin Resistance. J. Diabetes Res..

[60] Mohammed I., Hollenberg M.D., Ding H., Triggle C.R. (2021). A Critical Review of the Evidence That Metformin Is a Putative Anti-Aging Drug That Enhances Healthspan and Extends Lifespan. Front. Endocrinol..

[61] Flowers E., Ramírez-Mares J.-D., Velazquez-Villafaña M., Rangel-Salazar R., Sucher A., Kanaya A.M., Aouizerat B.E., Monroy M.L.L.d.l.V. (2021). Circulating microRNAs associated with prediabetes and geographic location in Latinos. Int. J. Diabetes Dev. Ctries..

[62] Aroda V.R., Ratner R.E. (2018). Metformin and Type 2 Diabetes Prevention. Diabetes Spectr..

[63] Hoskin M.A., Bray G.A., Hattaway K., Khare-Ranade P.A., Pomeroy J., Semler L.N., Weinzierl V.A., Wylie-Rosett J., Diabetes Prevention Program Research Group (2014). Prevention of Diabetes Through Lifestyle Intervention: Lessons Learned from the Diabetes Prevention Program and Outcomes Study and its Translation to Practice. Curr. Nutr. Rep..

